# Supplementary material for: Neuronal network remodeling and Wnt pathway dysregulation in the intra-hippocampal kainate mouse model of temporal lobe epilepsy
Source: PLoS One. 2019 Oct 9;14(10):e0215789. doi: 10.1371/journal.pone.0215789 (PMC6785072; doi:10.1371/journal.pone.0215789)
Supplement: S1 Table — Transcriptional data of Wnt genes 3 days after seizure induction, demonstrating fold-change in gene expression in seizure group compared to control. (DOCX) [file pone.0215789.s001.docx]

| Ipsilateral Dorsal (fold change 3d after seizure) | | | |
| --- | --- | --- | --- |
| Gene | Mean | Standard Deviation | P value |
| DKK-1 | 1.051065 | 0.233757289 | 0.895419 |
| WLS | 0.980918 | 0.156286268 | 0.805228 |
| WNT3 | 0.355371 | 0.119881334 | 0.00367 |
| WNT5A | 0.475751 | 0.119987528 | 0.010022 |
| WNT5B | 1.58807 | 0.11944974 | 0.012298 |
| WNT7A | 0.752495 | 0.071764906 | 0.075931 |
| WNT7B | 1.960959 | 0.217168832 | 0.00042 |
| WNT8B | 0.850384 | 0.324755451 | 0.476371 |
| WNT9A | 0.339899 | 0.077982371 | 0.000757 |
|  |  |  |  |
| Ipsilateral Ventral (fold change 3d after seizure) | | | |
| Gene | Mean | Standard Deviation | P value |
| DKK-1 | 0.563279 | 0.142733112 | 0.028256 |
| WLS | 0.974213 | 0.083485818 | 0.743541 |
| WNT3 | 0.830654 | 0.136067511 | 0.329183 |
| WNT5A | 0.611471 | 0.069289358 | 0.001156 |
| WNT5B | 1.09919 | 0.142765995 | 0.547533 |
| WNT7A | 0.496876 | 0.062598168 | 0.001811 |
| WNT7B | 1.426001 | 0.188268891 | 0.025978 |
| WNT8B | 1.285691 | 0.356024588 | 0.665836 |
| WNT9A | 0.490241 | 0.023282937 | 0.003759 |
|  |  |  |  |
| Contralateral (fold change 3d after seizure) | | |  |
| Gene | Mean | Standard Deviation | P value |
| DKK-1 | 0.477355 | 0.117410766 | 0.009379 |
| WLS | 1.02556 | 0.089125357 | 0.72053 |
| WNT3 | 0.685696 | 0.110569671 | 0.025802 |
| WNT5A | 0.639391 | 0.050883245 | 0.001357 |
| WNT5B | 1.066152 | 0.043853383 | 0.604828 |
| WNT7A | 0.551116 | 0.078158515 | 0.001959 |
| WNT7B | 1.299894 | 0.124136587 | 0.020855 |
| WNT8B | 1.141534 | 0.29222388 | 0.70239 |
| WNT9A | 0.184151 | 0.023730855 | 1.25E-05 |
